# A281 CASE REPORT: STEATOTIC LIVER DISEASE DIAGNOSED IN A 24-YEAR-OLD FEMALE WITH RETT SYNDROME

**DOI:** 10.1093/jcag/gwad061.281

**Published:** 2024-02-14

**Authors:** L G Albino, B Halloran, A Thiesen, A Adatia, V Dong, C Moctezuma Velázquez

**Affiliations:** University of Alberta Faculty of Medicine & Dentistry, Edmonton, AB, Canada; University of Alberta Faculty of Medicine & Dentistry, Edmonton, AB, Canada; University of Alberta Faculty of Medicine & Dentistry, Edmonton, AB, Canada; University of Alberta Faculty of Medicine & Dentistry, Edmonton, AB, Canada; University of Calgary Cumming School of Medicine, Calgary, AB, Canada; University of Alberta Faculty of Medicine & Dentistry, Edmonton, AB, Canada

## Abstract

**Background:**

Rett syndrome (RTT) is a rare, severe neurodevelopmental disorder seen almost exclusively in females that is caused by spontaneous loss-of-function mutations in the methyl CpG binding protein 2 (MECP2) gene. Girls with RTT typically demonstrate normal development for the first 6-18 months of life before displaying evidence of variable levels of developmental milestone regression and physical disability. Although perturbed lipid metabolism in the nervous system is the main contributor to the pathophysiology of RTT, mutations to the MECP2 gene also contribute to disease burden by causing perturbations to peripheral systems, leading to consequences such as dyslipidemia (DLD) and steatotic liver disease (SLD). Although DLD has been described in human cell lines and girls with RTT, SLD has only been described in RTT mouse models.

**Aims:**

This report aims to present a case of a patient with RTT who was diagnosed with SLD on liver biopsy in the context of DLD but without insulin resistance or other comorbidities associated with metabolic syndrome.

**Methods:**

Retrospective review of one patient.

**Results:**

A 24-year-old woman with genetically proven RTT (T158M point mutation to MECP2) complicated by several physical disabilities was seen at a tertiary care center clinic for evaluation of abnormal liver enzymes (ALT 163, AST 51, ALP 258, total bilirubin 6). Abdominal ultrasound demonstrated moderately echogenic liver parenchyma consistent with hepatic steatosis. Additional investigations for an etiology were unremarkable and the patient had no history of alcohol misuse or the use of new medications or supplements. Regarding risk factors for SLD, her homeostatic model assessment for insulin resistance (HOMA-IR) score was 1.8, hemoglobin A1c was 4.8%, and her lipid profile demonstrated elevated triglycerides and LDL, in keeping with DLD. The decision was made to proceed with a liver biopsy. This demonstrated moderate steatosis and no ballooning, categorized as grade A1 and stage F1.

**Conclusions:**

This case demonstrates that RTT patients have similar lipid metabolism perturbations to those seen in MECP2-null mouse models, resulting in similar metabolic abnormalities such as DLD and SLD. The absence of insulin resistance and other comorbidities associated with metabolic syndrome further support these perturbations are caused by a pathophysiologic mechanism specific to RTT. This demonstrates the importance of screening RTT patients for DLD and SLD despite their young age to detect these conditions before complications arise. It also demonstrates that MECP2-null mice can be used as reliable models to identify future therapies for RTT, such as ketogenic diet, ω-3 PUFAs supplementation, and lipid-lowering statin therapy.

**Funding Agencies:**

None

